# Behavior Assessment of Children in Dental Settings: A Retrospective Study

**DOI:** 10.5005/jp-journals-10005-1078

**Published:** 2011-04-15

**Authors:** Arun Sharma, Rishi Tyagi

**Affiliations:** 1 Professor, Department of Pedodontics and Preventive Dentistry, ITS Centre for Dental Studies and Research Muradnagar, Ghaziabad, Uttar Pradesh, India; 2 Reader, Department of Pedodontics and Preventive Dentistry, University College of Medical Sciences, New Delhi, India

**Keywords:** Behavior assessment, Behavior evaluation, Behavior management.

## Abstract

In children, dental anxiety and fear of dental treatment have been recognized as a source of problems in patient management for many years, which can affect the quality of care.

**Aims :** The aim of the study was to assess the behavior of children during their dental visit and to determine the effect of behavior management techniques on children.

**Materials and Methods :** A retrospective study was done by analyzing the records of 328 children to assess their behavior during dental visits. Behavior assessment was done using Frankl’s behavior rating scale. All children were exposed to live modeling and tell-show-do behavior management technique.

**Statistical analysis :** The analysis was carried out using SPSS version 10. The comparison between visits was carried out by applying Friedman test.

**Results and conclusion :** Children showed improvement in their behavior with every subsequent visits. Proper assessment of children’s behavior helps the dentist to execute a required treatment plan in the most appropriate manner. Techniques like live modeling and tell-show-do are very effective in modifying a child’s behavior.

## INTRODUCTION

Assessment of children based on their behavior is one of the most important skills for a pediatric dentist.^1^ The major aspect of child management in the dental care is managing dental anxiety and fear as it is considered to be the main barrier for successful completion of dental treatment. Children are known to have unfound fear and anxiety. The preschool children do not have the ability to understand a situation. They are in the very early stage of behavior development and little concern with the effect of their behavior on the observer. As the child enters the school, the child initiates the process of socializing and learning the conforming boundaries of behavior. Invariably, the pediatric dentists prefer to use pharmacological measures to achieve a required level of cooperation from very young children (preschool).

Many behavior rating scales are available to assess and evaluate the behavior of a child on each dental visit. Frankl et al classified child’s behavior into four groups according to the child’s attitude and cooperation or lack of cooperation during dental treatment.^2^ This classification is known as Frankl behavior rating scale, which is one of the most reliable tools developed for behavior rating of children in dental sittings.

The child’s behavior on dental visit can be influenced by variables like age, parental behavior, parental anxiety, past medical and dental history, awareness of their dental problem, type of dental settings, behavior management and procedural techniques followed by the dentist.^3^

Several techniques for managing children in the dental office have been developed to modify a child’s behavior. The nonpharmacologic tell-show-do technique which consists of verbal explanation of the procedure to the patient, demonstration for the patient of the (visual, auditory and tactile) aspects of the procedure and completion of the procedure, remains the most commonly used technique in pediatric dentistry.^4,5^

Modeling, nonpharmacologic behavior management technique was described by Bandura in 1967 as a process of acquiring behavior through observation of a model. Observing a peer (whether live/filmed) successfully undergoing dental treatment is effective in reducing children’s fear and anxiety about the dental treatment.^6-8^

The aim of the study was to analyze and assess the behavior of children during their dental visit and to determine the effect of behavior management techniques (tell-show-do and live modeling) on children.

## MATERIALS AND METHODS

The study was conducted in the Department of Pedodontics and Preventive Dentistry, ITS Center for Dental Studies and Research, Ghaziabad, India, to assess the behavior of children during their dental visits and to determine the effect of behavior management techniques on children. The study was done by analyzing the records of 328 children between the age group of 2 to 14 years who had undergone dental treatment. The treatment rendered varied from oral prophylaxis to restorative pulp therapy and extractions.

### Selection Criteria

The records of children undergone behavior assessment by operator for three dental visits to the department of pedodontics for dental treatment were included for the study.

Children were divided in four groups based on their ages. Group I (preschool) comprised of 17 children between age group of 2 and 3 years. Group II (kindergarden) comprised of 63 children between the ages of 4 and 5 years. Group III (primary school) comprised of 191 children between the age group of 6 and 10 years. Group IV (middle school) comprised of 57 children between the age group of 11 and 14 years.

During each visit (three visits), child had undergone assessment of behavior, i.e. pretreatment, during treatment and post-treatment. Behavior was recorded: (i) Before start of treatment (pretreatment) at the play therapy room, (ii) when treatment was rendered and (iii) post-treatment before relieving the patient from the department. Behavior assessment was done using Frankl’s behavior rating scale.^2,3^

*Definitely negative:* Refusal of treatment, crying forcefully, fearful, or any other overt evidence of extreme negativizm.

*Negative:* Reluctant to accept treatment, uncooperative, some evidence of negative attitude but not pronounced (sullen, withdrawn).

*Positive:* Acceptance of treatment, at times cautious, willingness to comply with the dentist, at times with reservation, but patient follows the dentist’s directions cooperatively.

*Definitely positive:* Good rapport with the dentist, interested in the dental procedures, laughing and enjoying.

Behavior was assessed by postgraduate students who were trained in behavior rating in the beginning of their curriculum. All children were exposed to an interaction with the operator in the play therapy room/reception area and then exposed to a positive live model in the dental clinic and then tell-show-do measure/technique was used on the dental chair.

The analysis was carried out using SPSS version 10. For statistics analysis, numerical value was assigned to Frankl’s behavior rating scale (Definitely negative-0, negative-1, positive-2, definitely positive-3). The comparison between visits was carried out by applying Friedman test and p-value < 0.05 was taken as significant.

## RESULTS

The results are summarized in Tables 1 to 4.

**Table 1:** **Table 1:** Behavior assessment for preschool children (group I)

		*Visit 1*						*Visit 2*						*Visit 3*					
		*pre-treatment*		*treatment*		*post-treatment*		*pre-treatment*		*treatment*		*post-treatment*		*pre-treatment*		*treatment*		*post-treatment*	
N = 17																			
(- -) 0		6 (35.29%)		7 (41.17%)		10 (58.82%)		2 (11.76%)		4 (23.52%)		4 (23.52%)		2 (11.76%)		2 (11.76%)		2 (11.76%)	
(-) 1		2 (11.76%)		4 (23.52%)		1 (5.88%)		3 (17.64%)		3 (17.64%)		2 (11.76%)		1 (5.88%)		1 (5.88%)		1 (5.88%)	
(+) 2		9 (52.94%)		6 (35.29%)		6 (35.29%)		12 (70.58%)		10 (58.82%)		11 (64.70%)		14 (82.35%)		14 (82.35%)		14	
(82.35%)																			
(++) 3		0		0		0		0		0		0		0		0		0	
Mean ± SD		1.18 ± 0.951		0.94 ± 0.899		0.76 ± 0.970		1.59 ± 0.712		1.35 ± 0.862		1.14 ± 0.870		1.71 ± 0.686		1.71 ± 0.686		1.76 ±	
0.562																			
Mean rank		1.65		1.53		1.50		2.12		2.06		2.06		2.24		2.41		2.44	
Chi-square test														11.200		15.200		16.188	
df(degree																			
of freedom)														2		2		2	
p-value														0.004		0.001		0.000	

## DISCUSSION

In our study in group I, six (35.29%) of the children were definitely negative on Frankl’s behavior rating scale and by the end of the 1st visit four more children had shown decreased level of cooperation and were graded as definitely negative. By the 2nd visit two (11.76%) children were graded as definitely negative, which is indicative of a better response in the 2nd visit of the same children. During treatment, two (11.76%) children showed a definitely negative behavior in the 3rd visit, this indicates lack of comprehension and understanding achieved by child. Number of children showing positive response had reasonably increased by the end of 3rd visit. A very small number of children did not cooperate for routine dental treatment. Simple counseling, tell-show-do and live modeling did have a great impact on younger children (preschool). This is especially important in our setup, as parents are increasingly apprehensive and less willing to allow the use of conscious sedation or undertake general anesthesia. In the age group of 4 to 5 years (group II) the number of children in definitely negative category were three (4.76%) and 13 (20.63%) were graded as negative. At the end of initial counseling and live modeling, five children become more uncooperative (definitely negative) and 17 children showed lack of the cooperation (negative). By the end of 3rd visit only one (1.58%) child was definitely negative and seven (11.11%) children were negative. In fact, for 4 to 5 years children these psychological measures are impressively effective and can create extremely good patient at this stage of life, as 16 (25.39%) were definitely positive by the end of the 3rd visit.

**Table 2:** **Table 2:** Behavior assessment for kindergarden children (group II)

		*Visit 1*						*Visit 2*						*Visit 3*					
		*pre-treatment*		*treatment*		*post-treatment*		*pre-treatment*		*treatment*		*post-treatment*		*pre-treatment*		*treatment*		*post-treatment*	
N = 63																			
(- -) 0		3 (4.76%)		4 (6.34%)		8 (12.69%)		0		1 (1.58%)		2 (3.17%)		0		0		1 (1.58%)	
(-) 1		13 (20.63%)		21 (33.33%)		17 (26.98%)		11 (17.46%)		17 (26.98%)		18 (28.57%)		7 (11.11%)		9 (14.28%)		7 (11.11%)	
(+) 2		47 (74.60%)		38 (60.31%)		38 (60.31%)		47 (74.60%)		37 (58.73%)		38 (60.31%)		47 (74.60%)		45 (71.42%)		39	
(61.90%)																			
(++) 3		0		0		0		5 (7.93%)		8 (12.69%)		5 (7.93%)		9 (14.28%)		9 (14.28%)		16	
(25.39%)																			
Mean ± SD		1.67 ± 0.568		1.54 ± 0.618		1.48 ± 0.715		1.90 ± 0.499		1.83 ± 0.661		1.73 ± 0.653		2.03 ± 0.507		2.00 ± 0.539		2.13 ±	
0.660																			
Mean rank		1.70		1.63		1.55		2.06		2.06		1.93		2.25		2.32		2.52	
Chi-square test														35.565		44.345		62.488	
df(degree of																			
freedom)														2		2		2	
p-value														0.000		0.000		0.000	

**Table 3:** **Table 3:** Behavior assessment for primary school children (group III)

		*Visit 1*						*Visit 2*						*Visit 3*					
		*pre-treatment*		*treatment*		*post-treatment*		*pre-treatment*		*treatment*		*post-treatment*		*pre-treatment*		*treatment*		*post-treatment*	
N = 191																			
(- -) 0		2 (1.04%)		2 (1.04%)		4 (2.09%)		1 (0.52%)		1 (0.52%)		1 (0.52%)		0		1 (0.52%)		1 (0.52%)	
(-) 1		6 (3.14%)		29 (15.18%)		35 (18.32%)		5 (2.61%)		11 (5.75%)		26 (13.61%)		2 (1.04%)		8 (4.18%)		14 (7.32%)	
(+) 2		183 (95.81%)		160 (83.76%)		152 (79.58%)		183 (95.81%) 173 (90.57%)				155 (81.15%)		183 (95.81%)		175 (91.62%)		158	
(82.72%)																			
(++) 3		0		0		0		2 (1.04%)		6 (3.14%)		9 (4.71%)		6 (3.14%)		7 (3.66%)		18 (9.42%)	
Mean ± SD		1.95 ± 0.266		1.83 ± 0.406		1.77 ± 0.466		1.97 ± 0.239		1.96 ± 0.330		1.91 ± 0.456		2.02 ± 0.204		1.98 ± 0.316		2.02 ±	
0.441																			
Mean rank		1.95		1.85		1.81		1.99		2.06		2.02		2.06		2.09		2.17	
Chi-square test														21.571		53.067		69.391	
df (degree of																			
freedom)														2		2		2	
p-value														0.000		0.000		0.000	

**Table 4:** **Table 4:** Behavior assessment for middle school children (group IV)

		*Visit 1*						*Visit 2*						*Visit 3*					
		*pre-treatment*		*treatment*		*post-treatment*		*pre-treatment*		*treatment*		*post-treatment*		*pre-treatment*		*treatment*		*post-treatment*	
N = 57															
(- -) 0		0		0		0		0		0		0		0		0		0	
(-) 1		3 (5.26%)		6 (10.52%)		7 (12.28%)		0		2 (3.50%)		3 (5.26%)		0		0		0	
(+) 2		43 (75.43%)		44 (77.19%)		46 (80.70%)		51 (89.47%)		50 (87.71%)		50 (87.71%)		48 (84.21%)		46 (80.70%)		46	
(80.70%)																	
(++) 3		11 (19.29%)		7 (12.28%)		4 (7.01%)		6 (10.52%)		5 (8.77%)		4 (7.01%)		9 (15.78%)		11 (19.29%)		11	
(19.29%)																	
Mean ± SD		2.14 ± 0.480		2.02 ± 0.481		1.95 ± 0.440		2.11 ± 0.310		2.05 ± 0.350		2.02 ± 0.353		2.16 ± 0.368		2.19 ± 0.398		2.19 ±	
0.398																	
Mean rank		2.01		1.89		1.84		1.96		1.95		1.95		2.04		2.16		2.21	
Chi-square test														1.750		14.000		22.286	
df (degree																			
of freedom)														2		2		2	
p-value														0.417		0.001		0.000	

In our study, children showed improvement in their behavior on subsequent visits. Frankl studied the cooperative behavior of 3 to 5 years old children during dental visit and concluded that cooperative behavior increases on second visit.^2^ Howitt and Stricker also concluded in their study that child’s arousal level was reduced as he gained experience in the dental situation.^9^ Venham and Cipes have also shown that the behavior of children improves in subsequent dental visits.^10^ The improvement during subsequent visits suggests that the experience gained by the child during previous visits helped the child to recognize the nonthreatening aspects of the visits and to deal with stressful dental procedures.

In group III between the age group of 6 to 10 years, the children are grown up and expected to understand and cooperate during dental treatment. However, we had four (2.09%) children showing definitely negative behavior during the end of 1st visit, and by the end of 3rd visit one (0.52%) child showed definitely negative behavior, although we have not assessed their family background and reason for the uncooperative behavior. It is possible to create positive attitude in this age group and achieve all possible treatment without the use of conscious sedation or general anesthesia. The gross reason for uncooperative behavior could be due to pain at the time of treatment. Corkey and Freeman have reported that dental anxiety begins to decrease by 6 to 7 years, with most children being able to cope with dental situations at that age.^11^ Holst and Crossner,^12^ and Klingberg et al^13^ have also established that dental anxiety was more pronounced in younger children (4-6 years) as compared to older children (9-11 years). Differences in child’s reaction to dental treatment according to age have been well-established.^14,15^

Age group beyond 10 years (group IV) shows a uniform positive behavior and willingness to cooperate and undergo necessary treatment. All negative images and thoughts are erased by the 3rd dental visit.

It is extremely important that child must be given due consideration on there visit to dental department, and simple behavior management techniques can have profound effect in achieving our goals to impart good oral health and the positive attitude towards dentistry. Pediatric dentist frequently face behavior management problems and continue to seek safe, cost-effective techniques for managing difficult children. Parental acceptance of behavior management technique used by pediatric dentist is another concern. Parents reported a significant preference for noninvasive reinforcement techniques instead of sedation and restrains.^16^ General principles were established to gauge the validity of behavior management techniques at the conference of the American Academy of Pediatric Dentistry in 2003: (a) Effectiveness—the potential of the technique to manage children’s behavior in the dentist’s office, (b) Social validity— acceptance of the technique by parents as well as public perception of the technique, (c) Risk associated with the technique, (d) Cost—time spend practicing the technique and cost of any materials and equipment used.^17^

Modeling (model learning) has been suggested as a method for introducing the child to dentistry.^6,18^ In this approach, the ability of the child to imitate others is used and by imitating, the child learns complex behavior patterns. Two forms of modeling, live and filmed, are effective in reducing children’s fear and anxiety about dental treatments and promoting adaptive behavior.^8,19^ Tell-show-do method is still considered the technique with which dentists and parents are most comfortable.^17,20^

In our study, these two behavior management techniques were effective in reducing child’s fear and anxiety as most of the children in our study were showing cooperative behavior by the end of 2nd or 3rd visit. The methods of behavior management should be designed, so that the child willingly returns to the clinic for regular check-ups through out the childhood years and into adult life with no reluctance. The use of pharmacological agents in the management of fearful or anxious child may help to achieve the treatment plan, but these approaches should never be seen as an alternative to manage the uncooperative child patient because of time constraint on the part of the dentist and should only be employed when absolutely indicated.

## CONCLUSION

Assessment of behavior is the most important tool in the hands of the dentist. This helps the dentist to execute required treatment plan in the most appropriate manner in children. Simple techniques like preparation of the child in nondental settings, like a play therapy room or a play area can be instrumental in instilling a positive behavior in a child. Techniques like live modeling and tell-show-do are very effective in achieving treatment goals in all age groups.
